# Using Personal Genomic Data within Primary Care: A Bioinformatics Approach to Pharmacogenomics

**DOI:** 10.3390/genes11121443

**Published:** 2020-11-30

**Authors:** Rick Overkleeft, Judith Tommel, Andrea W. M. Evers, Johan T. den Dunnen, Marco Roos, Marie-José Hoefmans, Walter E. Schrader, Jesse J. Swen, Mattijs E. Numans, Elisa J. F. Houwink

**Affiliations:** 1 Coöperatie 4LifeSupport Europa u.a., 2321 JW Leiden, The Netherlands; 2 4MedBox, 2321 JW Leiden, The Netherlands; 3 Health, Medical and Neuropsychology Unit, Institute of Psychology, Faculty of Social and Behavioural Sciences, Leiden University, 2333 AK Leiden, The Netherlands; j.tommel@fsw.leidenuniv.nl (J.T.); a.evers@fsw.leidenuniv.nl (A.W.M.E.); 4 Departments of Human Genetics and Clinical Genetics, Leiden University Medical Center, 2333 ZA Leiden, The Netherlands; ddunnen@humgen.nl; 5 Human Genetics Department, Leiden University Medical Center, 2333 ZA Leiden, The Netherlands; m.roos@lumc.nl; 6 Schluss, 1013 KS Amsterdam, The Netherlands; m-j@schluss.org; 7 General Practitioner, 2313 AX Leiden, The Netherlands; weslink@wxs.nl; 8 Department of Clinical Pharmacy and Toxicology, Leiden University Medical Center, 2333 ZA Leiden, The Netherlands; J.J.Swen@lumc.nl; 9 Department of Public Health and Primary Care (PHEG), Leiden University Medical Centre, 2333 ZA Leiden, The Netherlands; M.E.Numans@lumc.nl (M.E.N.); E.J.F.Houwink@lumc.nl (E.J.F.H.); 10 National eHealth Living Lab (NELL), 2333 ZD Leiden, The Netherlands

**Keywords:** pharmacogenomics, primary care, genomics, research, ethics, Personal Genetic Locker, personalized medicine

## Abstract

One application of personalized medicine is the tailoring of medication to the individual, so that the medication will have the highest chance of success. In order to individualize medication, one must have a complete inventory of all current pharmaceutical compounds (a detailed formulary) combined with pharmacogenetic datasets, the genetic makeup of the patient, their (medical) family history and other health-related data. For healthcare professionals to make the best use of this information, it must be visualized in a way that makes the most medically relevant data accessible for their decision-making. Similarly, to enable bioinformatics analysis of these data, it must be prepared and provided through an interface for controlled computational analysis. Due to the high degree of personal information gathered for such initiatives, privacy-sensitive implementation choices and ethical standards are paramount. The Personal Genetic Locker project provides an approach to enable the use of personal genomic data in primary care. In this paper, we provide a description of the Personal Genetic Locker project and show its utility through a use case based on open standards, which is illustrated by the 4MedBox system.

## 1. Personalized Medicine and Pharmacogenomics

### 1.1. Personalized Medicine

Personalized medicine is a term used for tailoring medication to the individual. Personalized medicine involves the physician considering family history and pharmacogenetics in daily practice. Access to information about an individual’s genetic and pharmacogenetic makeup would therefore provide another source of personalized data. It would enable doctors to better define the nature of a disease and find the most effective treatment for a particular patient, in order to avoid ineffectiveness and unexpected side effects. With the help of pharmacogenetic studies, physicians will be able to administer treatment regimens that are personalized and adapted to each person’s genetic makeup. Furthermore, they will be also able to pre-symptomatically diagnose and discuss individualized treatment plans.

### 1.2. Pharmacogenomics

Pharmacogenomics is widely recognized as one of the first clinical applications of personalized medicine. It aims to optimize drug treatment by personalizing the dose and drug selection based on a better understanding of the genetic variation that is causal for the variability in drug response, typically via alterations in a drug’s pharmacokinetics (e.g., metabolism) or via modulation of a drug’s pharmacodynamics (e.g., the drug target). After decades of discovery, the field of pharmacogenetics is moving towards clinical implementation, thereby providing a cornerstone for personalized medicine. Surveys show that both pharmacists and physicians have high expectations of utilizing pharmacogenetics to guide prescribing and dispensing drugs [1,2]. To help general practitioners and pharmacists with the interpretation of a pharmacogenomic test result, the Dutch Pharmacogenetics Working Group (DPWG) and the Clinical Pharmacogenetics Implementation Consortium (CPIC) have developed pharmacogenomics (PGx) guidelines [3]. Together, these consortia have evaluated over 100 gene–drug interactions and provide therapeutic recommendations for ~50 drug–gene pairs. Whilst the availability of these guidelines presents a major step towards clinical applications of pharmacogenomics, the applicability is limited by the relatively low numbers of patients that have their pharmacogenetic test results. Recently, it was estimated that should we have known the pharmacogenetic profile of all Dutch inhabitants, we would have adjusted the dose or switched to another drug in 5.4% of all new drug prescriptions [4]. The first interesting attempts to implement pharmacogenomics into a decision support system have been made by the industry and will be taken into account [5].

### 1.3. Combining Family History and Genetics

A family history of asthma, chronic obstructive pulmonary disease (COPD), diabetes, cardiovascular diseases or various types of cancers (including prostate cancer, ovarian cancer, melanoma, breast cancer and colon cancer) leads to a relative risk for these diseases that is two to five times higher than that of people without a positive family history, irrespective of known genetic associations (e.g., *BRCA1/2*) [6]. When multiple family members are affected with these common diseases, and when this occurs at a young age, the relative risk increases further [7,8]. Clinical phenotyping by taking the family history is therefore a useful, low-cost and easy tool for pre-symptomatic risk assessment for multiple common chronic diseases (e.g., asthma, COPD, cardiovascular diseases, cancer, diabetes) in daily primary care practice, reinforced by the role of general practitioners (GPs) as family doctors. A family history could open possibilities for early primary and secondary prevention of these diseases and their monogenetic disease equivalents (e.g., long QT syndrome, breast cancer caused by *BRCA1/2* mutations, Maturity onset diabetes of the young (MODY) subtypes) and could also be used to find, inform and treat unaffected family members pre-symptomatically [9]. Clinicians recognize an urgent need for competence in recording family history data and in the registration of a family history questionnaire in the electronic health record (EHR). However, a lack of availability of relevant data and knowledge regarding genetics, pharmacogenetics and logistics towards registration, hampers implementation [10]. Nevertheless, GPs agree that taking a family history using a validated questionnaire could be an important tool in good clinical practice, as it allows for familial risk stratification and the identification of hereditary conditions [7,10,11,12]. In addition, it was suggested that a validated online family history questionnaire could aid in the decision-making process (decision support systems) surrounding the consultation of a clinical geneticist for further diagnosis in accordance with current clinical genetics referral guidelines [13]. 

Recently, a validated family history questionnaire was developed and published and could therefore help integrate genetics into the EHR, leading to the rapid operationalisation of readily available genetic knowledge in daily practice and clinical research, consequently improving medical care [6]. Taking into account that specific subtypes of certain diseases, i.e., COPD, may benefit from a certain treatment while other subtypes of the same disease may have a negative effect from that treatment [14], it is of the utmost importance to perform such phenotypical determinations. This process of phenotypical determination helps identify the patients that need to be genotyped to achieve a better understanding of the disease subtypes.

Primary care has the opportunity to promote personalized medicine through integration of genomic medicine, family history, pharmacogenetic and genetic test results. Risk assessment for certain diseases before they occur, and personalized proactive treatments, may become available. EHRs need to be reimagined to make disease risk assessment possible, with an opportunity to embark upon proactive interventions and timely referrals to the department of clinical genetics, to implement accurate diagnostics and even reconciliation of choice of medication or dosage of certain medication using the pharmacogenetic profile of the individual patient in daily practice [15,16].

## 2. The Data Gap

### 2.1. Providing Data to the Primary Care Professional

As individuals start to collect their own data (by using devices such as smart watches for heart monitoring or DNA testing/whole genome sequencing services), they wish to see the result applied in their daily lives [17,18,19]. Unfortunately, these types of data never reach primary care professionals, since there are no mechanisms to store the data collected by the patient, and there is no system for viewing, interpreting, and sharing these data. Even when an individual tries to provide these data directly to a primary care professional, the professional cannot use it, as the quality cannot be evaluated. The standard deviations of devices used is unknown and it is not known whether data were modified, etc. On top of that, any new data source requires primary care professionals to spend time and effort on understanding and interpreting the data, which reduces time available to provide care to those that need it.

Although the added benefits of genetic and pharmacogenetic information about an individual are generally acknowledged, the full implementation of a widely distributed system to facilitate genetic and pharmacogenetic knowledge distribution towards primary care professionals is currently absent. Many obstacles are encountered upon implementation of such a system. 

### 2.2. Contextual Knowledge

To provide the individual with the right care, contextual information is essential. Combining genotypical data with phenotypical, psychological, well-being and family history data creates a holistic approach to the health of the individual, now and in the future. 

This holistic approach is not only necessary for providing the right primary care for the individual, as it can also be used for research purposes. A researcher requesting data for their research from the individual can request contextual data, if present, without going through the process of collecting the data themselves. Such a research process could drastically reduce the cost and time for data collection for research. If the results of the research are additionally returned to the individual, the results create more contextual knowledge about that individual and could be used in primary care or re-used in subsequent research studies.

## 3. Research and Privacy

### 3.1. Direct Impact between Primary Care and Academic Research

When genetic data are collected in a way that prepares data for reuse (e.g., by implementing Findable, Accessible, Interoperable, and Reusable (FAIR) principles [20]), data are immediately available when required. We could, for example, act quickly, cheaply, and effectively in response to public health emergencies, such as the COVID-19 pandemic. 

If genetic data for a patient have already been generated (with appropriate consent and access conditions that can be assessed automatically), we could readily link personal data to the study of possible genetic associations for that individual upon exposure to the COVID-19 virus, the specific attributes of the viral genome, and the consequences for the health of the affected individual after treatment.

For a long time, data collection has been an integral part of the scientific method. Following the scientific method, one establishes a hypothesis and a method for collecting the required data. The data are then collected using the described methods, and results and conclusions are drawn. With the advent of data science and big data, the scientific method has evolved to allow more data-driven approaches. In the last decade, genetic research has become more data driven with the introduction of cheaper, high-throughput techniques, providing realistic opportunities for including DNA sequencing in primary and secondary care, without a specific (scientific) hypothesis. Often, individuals opt to buy commercial sequencing services and generate their own data. These vast and growing collections of biomedical data can be a critical resource for scientific research if quality is assured and data are collected without bias. The availability of these data also requires a shift in thinking within scientific research: data are already collected, so computational methods involve simply mining these data using routine and reproducible protocols, in order to answer a hypothesis. 

To increase the adoption rate of such an intricate and complex system, one must actively search for feedback from primary care professionals, researchers, and also the general public (who are both patients and healthy individuals who may become patients). 

### 3.2. Genomic Data, Privacy and Ethics

The increasing need for sharing genomic data for research purposes escalates the tension between genomic privacy and openness [21]. As genomic data cannot be anonymized while still keeping the information intact, it is a centre piece of controversy. 

On the one hand, the information is needed to provide medical care or perform genomic research; on the other hand, the information is directly correlated to an individual and therefore is private information and falls under the General Data Protection Regulation (GDPR). Of particular interest is the concept of the “personal health train”, where FAIR principles at source enable data to be visited on site for analysis, not moved [22,23]. Applied to Personal Genetic Lockers, this means that lockers are visited with an analysis request. Access restrictions described in FAIR metadata are subsequently assessed to negotiate access, and then the analysis takes place inside a safe environment in or associated with the locker. Only unidentifiable results can subsequently “leave” the locker. Multiple lockers can be visited for federated analysis in this way. 

One of the main focus points of a system that facilitates the understanding of the contextual knowledge around an individual is a solid ethical foundation on which it is built upon. Not one single organization or one single group of people may be the only ones to discuss the ethical perspective of such a system. The Ethical, Legal, and Social Implications (ELSI) program by the United States National Institute of Health has already taken steps to pave the way for the ethical implications of genetic research and the GDPR by the European Union has provided a foundation for the ethical handling of personal information. These and many more ethical standpoints from the perspective of a European citizen need to be discussed in a transparent manner.

## 4. Personal Genetic Locker

Pharmacogenomics is a rapidly growing field in which studying the genetic differences influences the variability of individual patient responses to drugs and aims to distinguish responders from non-responders and predict those in whom toxicity from drugs will be more prevalent. It can be regarded as the 21st century’s way to prescribe drugs—the right drug to the right patient at the right dose. The frequently perceived hurdle for the clinical uptake of pharmacogenomics is the availability of guidelines translating pharmacogenomic test results into clinical actions for individual patients [15,24]. Supplying bioinformatics analysis to the care provider through a clinical decision support system will assist them in the decision-making process and comply to new guidelines, while taking the ELSI into account.

As an answer to this growing need for guidelines, the Personal Genetic Locker (PGL) project is conceived. This project proposes an Information and Communication Technology (ICT) infrastructure that allows individuals to access their own health data, understand the health implication, add their own health-related information and consent to, or prevent, sharing with treating physicians and/or use for research projects, this is named a personal locker. Although a personal locker can be used for a wide range of health data, the PGL will define standards and prototypes for a safe environment where individuals can store, view, and interpret their personal genetic data [25], which will be openly published when defined. Personal genetic data are defined by the PGL as any and all data gathered from the genome of the individual. This choice has been made since the information present in an individual’s DNA is immediately relevant for medical treatment. For implementation purposes, a real life (pharmacogenomics) use case is used, from which social, ethical, and technical issues can be discussed and standards can be defined with. Within the PGL project, there are two schools of thought: the first is a more centralised approach, where patients can access their information stored at an institute and control access to their data to primary care providers; the second is a decentralised approach, where the patient/individual can choose where the data are stored and provide access to primary care providers when needed. Although the two schools have a different approach, they have roughly the same challenges. Depending on legal requirements and ethical considerations, the Personal Genetic Locker will also support management of data for which responsibility is shared between groups of individuals.

From a technology point of view, the Personal Genetic Locker concept aligns with initiatives such as MyData [26] and Single responsibility, Open-closed, Liskov substitution, Interface segregation and Dependency inversion (SOLID) [27] that aim to provide principles and a technological framework for individuals to gain control over personal digital data. Partners in the PGL project, such as 4MedBox, the Schluss foundation, and Whitebox, provide prototypes that allow patients, care providers and researchers to become acquainted with the concept. Furthermore, the requirements for the efficient use of pharmacogenomic data imply implementing procedures and technology that prepare data for controlled, computational analysis. The FAIR principles provide some guidance [20] on the digital exchange of data with researchers: a “FAIR” locker describes, in a computer readable language, what it is about and how data are findable, accessible under transparent conditions (i.e., not open data!), interoperable, and reusable. As adoption of FAIR principles increases, personal genetic lockers can become part of a larger ecosystem of FAIR data resources.

As discussed in the previous section, a solid ethical foundation needs to be set in place to have such a system work properly. Since the PGL project team defined that such a foundation cannot be created by a single project/organisation, it is actively searching for European groups and organisations to discuss with and actively participate in taking the next steps in shaping the ethical foundation, for instance via the MyData global organisation [28], the European Joint Programme Rare Diseases [29] or the Digital House of Europe [30]. 

Future publications will go into more detail about the standards defined and the different prototypes that are being created in the PGL project.

### 4.1. First Use Case for Implementation

For this use case, we use the case of an individual (scientist) who performed DNA sequencing on his own DNA (twice) and bought several commercial DNA health tests. When presenting these DNA data (including annotations) to his general practitioner for making decisions on which drugs to prescribe, they were dismissed, even when specific results were corroborated across multiple samples. 

The data were considered untrustworthy and unreliable because they had not been generated in a standard health care system, even though the commercial analysis performed was more extensive and more detailed than any current standard procedure. If the data would have been accepted, there was no easy way for the primary care professional to analyse the data and confirm results before deciding on which medication would be best. In addition, interpreting genomics and pharmacogenomics data is a complex task that requires specialized training. Many GPs currently lack this knowledge and therefore cannot make the best use of these data.

The result is that, despite scientific research making breakthrough after breakthrough in understanding and applying genetic information, many obstacles remain in using the genetic information in primary care. As a consequence, the promise of personalizing treatments and reducing health care costs significantly by using prognostic genetic information is far from being realized.

The PGL applies this use case to initiate the discussion and to define standards. 

### 4.2. 4MedBox

**Figure 1:**
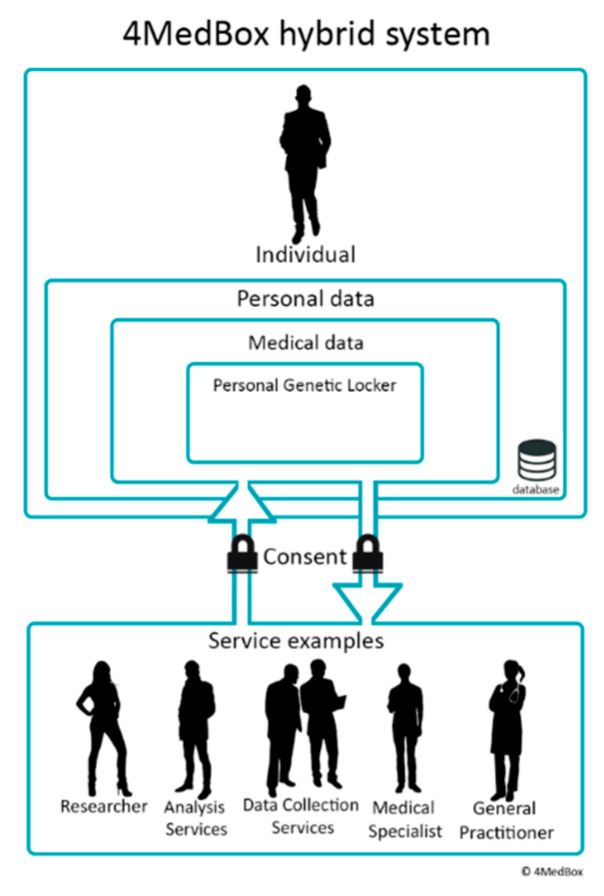
The interaction in the 4MedBox system between an individual and examples of possible medical services an individual can use. The image visualizes control over the data by the individual by giving consent.

An example of a system created by a member of the PGL project is the 4MedBox system, which started implementing the first use case as a starting point for the development of a PGL. The 4MedBox system defined that the individual/patient is the starting point (the decentralized approach) to whom different services are provided by different service providers, such as general practitioners, pharmacists, data collection services (i.e., DNA sequencing services), analysis services (i.e., genetic data mapping to pharmacogenetic datasets), etc. In the 4MedBox system, all meta-data (the decision-making process, the guidelines/standards, the decision that is made, the notes/reflections created by the care provider (the interpretation of the data), etc.) are stored with a direct correlation to and alongside the collected data. This ensures both contextual knowledge about the individual as well as the reliability of the data. This information and the vast amount of measurement data that are collected in clinical practice, by individuals themselves, and analysis performed on the data, can directly benefit academic research when data and gathering method(s) are defined and accessible for academic research. In Figure 1, an abstract visualization is shown of the individual having full control over what data are or are not viewed or analysed by any service or party.

## 5. Adoption

The general public usually recognizes the benefits of genetic research. However, a number of concerns and potential participant risks have been raised regarding genetic research [31,32]. In personalized genomic medicine, major concerns regarding the ethical, legal, and social implications (ELSI) involve topics such as consent, disclosure, data sharing, privacy, and confidentiality [33]. A focus group study, for example, concluded that participants felt generally positive about participating in genetic research, but they worried about sharing genetic data and opted for more transparency. This study showed a clear need for more information and education about genetic research to increase public understanding and to address potential concerns [32]. 

Since the individual’s needs and choices are the core of the PGL, we should involve future individual users in the developmental process as early as possible to match the PGL to the users’ needs and wishes and to take their concerns into consideration. Therefore, we aim to form a focus group study to evaluate future users’ needs and potential concerns and to formulate the required functionalities and characteristics of the PGL.

A focus group is a discussion-based interview [34]. The purpose of conducting a focus group is to explore and better understand how people think or feel about a certain issue or product. Focus groups can be explained by five typical characteristics: focus groups include (1) a small group of people, who (2) possess certain characteristics, (3) provide qualitative data (4) in a focused discussion (5) to help understand the topic of interest [35]. 

Within the PGL project, we aim to organize two separate focus group interviews: one including health care users and one including health care professionals. Both groups of individuals include future users of the PGL; however, the setting in which they will use the PGL will differ. Health care users will use the PGL in a private setting, for example, to manage and monitor their own health. Health care professionals, however, will use the PGL in a strictly professional setting to manage and monitor their patients’ health and to guide treatment. These different settings will probably affect how individuals feel and think about a PGL. In addition, private use will probably ask for different functionalities and characteristics compared to professional use. In the focus group study, we will include individuals representing both settings in order to get a complete overview of the required functionalities and to match the PGL to the users’ needs and wishes.

## 6. Implementation

### 6.1. Steps to Overcome Implementation Obstacles

As mentioned before, there are many obstacles to overcome before being able to call the implementation of such a system a success. The following obstacles, among others, will be addressed, some within the PGL project and some outside of the project:

- Language barriers for better adoption [36];
- Adoption of all stakeholders (patient, GP, medical specialist, etc.) within the health care systems [37] with clear definitions of roles and responsibilities [24];
- Diversifying research populations [38];
- Inclusion of minors, parental consent and when rights are transferred from parent to child [39];
- Creating an ethical foundation for the system;
- Providing storage for personal genetic data;
- Provide data visiting capabilities based on FAIR principles;
- Providing primary care professionals the means to interpret the genetic data of their patients [40];
- Technological standards to share across primary care and research all over Europe;
- Architecture for such a system.

These hurdles will be addressed in upcoming publications.

### 6.2. First Study

The first use case describes an individual that has their DNA data, but the primary care professional cannot use this information in daily practice. Using this first use case, the first study will be to find correlations between family history, clinical phenotyping, and genomic data within the PGL of an individual—to research the possibility of data re-use of sequenced DNA within primary care. This can be used as a foundation upon which the next study can be designed. The next study will analyse pharmacogenomic datasets and propose medication within primary care by mapping these to personal genomic datasets within the PGL of an individual.

## 7. Concluding Remarks and Future Directions

Genetics and especially pharmacogenetics are widely accepted as being beneficial for primary care, but guidelines for translating pharmacogenomic test results into clinical actions have yet to be conceived. In this article, we have discussed the aspects involved and proposed solutions for some of the steps that need to be performed to implement such a system successfully and the project that will take the first steps of creating standards. Next to technical and organisational implementation steps, we have discussed the ethical implications of such a system and our continuous search for more European organisations to create a solid ethical foundation for the system. The Personal Genetic Locker project aims to implement personal genomic data effectively in primary care.

In upcoming publications, the next steps of implementation, the proposed first research topic, and a more detailed description of the standards will be discussed.

### From Primary Care to Preventive Care

The above discussed methods to combine data for use in primary care can also benefit the individual before the need for primary care is there. Most healthcare systems are based on the premise that one needs healthcare when one is ill. This is a reactive approach to healthcare and in itself flawed, since one cannot provide preventive (proactive) healthcare interventions. Hospitals and physicians will, in some cases, even actively reject individuals who want to preventively perform genetic diagnostic tests. When these tests are then performed outside of the healthcare system, by commercial companies where ethical implications are not always adequately addressed, the primary care professionals are not equipped to retrieve and use these data. 

To lower costs in the healthcare system and increase quality of life, the healthcare system must become proactive and allow individuals to perform proactive diagnostic tests and analysis. This information will allow the individual to be better prepared and change behaviour before diseases are prevalent. For instance, it may become possible to:

- Use the pharmacogenetics profile to get the best medication;
- Know about risks for hereditary disorders and treat them early where possible;
- Perform a genetic health-risk assessment for personalized follow-up and where possible reduce risks early by lifestyle adjustments;
- As soon as one wants children, use it to compare with the partner’s DNA to identify possible risks of hereditary disorders for the children.

All only performed when the individual gives consent and following the highest ethical standards.
